# Supplementary material for: Single Cell Transcriptomic and Chromatin Profiles Suggest Layer Vb Is the Only Layer With Shared Excitatory Cell Types in the Medial and Lateral Entorhinal Cortex
Source: Front Neural Circuits. 2022 Jan 26;15:806154. doi: 10.3389/fncir.2021.806154 (PMC8826650; doi:10.3389/fncir.2021.806154)
Supplement: Supplementary file 14 [file Data_Sheet_1.ZIP › 6/knownResults.html]

6 - Homer Known Motif Enrichment Results


# Homer Known Motif Enrichment Results (6)

Homer *de novo* Motif Results  
Gene Ontology Enrichment Results  
Known Motif Enrichment Results (txt file)  
Total Target Sequences = 19747, Total Background Sequences = 29688

|  |  |  |  |  |  |  |  |  |  |  |  |
| --- | --- | --- | --- | --- | --- | --- | --- | --- | --- | --- | --- |
| Rank | Motif | Name | P-value | log P-pvalue | q-value (Benjamini) | # Target Sequences with Motif | % of Targets Sequences with Motif | # Background Sequences with Motif | % of Background Sequences with Motif | Motif File | SVG |
| 1 | C G T A T A C G T C G A A C T G A C T G C G T A C G T A T A C G A G C T T A C G | PU.1(ETS)/ThioMac-PU.1-ChIP-Seq(GSE21512)/Homer | 1e-832 | -1.917e+03 | 0.0000 | 4133.0 | 20.93% | 2134.6 | 7.19% | motif file (matrix) | svg |
| 2 | C T G A A G T C C G A T A G C T A T G C G T A C A C G T A T C G C A G T G C A T | Elf4(ETS)/BMDM-Elf4-ChIP-Seq(GSE88699)/Homer | 1e-738 | -1.699e+03 | 0.0000 | 6747.0 | 34.16% | 5046.9 | 17.01% | motif file (matrix) | svg |
| 3 | C G T A C T G A C G T A C T A G T C G A C T A G A C T G C G T A C G T A T A C G A G C T A T C G | SpiB(ETS)/OCILY3-SPIB-ChIP-Seq(GSE56857)/Homer | 1e-656 | -1.512e+03 | 0.0000 | 2426.0 | 12.28% | 975.4 | 3.29% | motif file (matrix) | svg |
| 4 | G C T A A G T C T A C G T G C A A T C G T C A G G C T A T C G A T C A G A G C T | ELF5(ETS)/T47D-ELF5-ChIP-Seq(GSE30407)/Homer | 1e-653 | -1.504e+03 | 0.0000 | 5041.0 | 25.52% | 3392.2 | 11.43% | motif file (matrix) | svg |
| 5 | T C G A T A G C T G C A A C T G A C T G C G T A C G T A C T A G G A C T T A C G | ETS1(ETS)/Jurkat-ETS1-ChIP-Seq(GSE17954)/Homer | 1e-638 | -1.469e+03 | 0.0000 | 6456.0 | 32.69% | 5005.2 | 16.87% | motif file (matrix) | svg |
| 6 | A T G C A G T C C T G A A G T C C G A T A C G T A G T C A G T C A C G T A T C G G A C T A C G T | Etv2(ETS)/ES-ER71-ChIP-Seq(GSE59402)/Homer | 1e-589 | -1.358e+03 | 0.0000 | 5662.0 | 28.67% | 4242.9 | 14.30% | motif file (matrix) | svg |
| 7 | T C G A T A G C G T C A A C T G A C T G C G T A C G T A C T A G A G C T T C A G | ERG(ETS)/VCaP-ERG-ChIP-Seq(GSE14097)/Homer | 1e-580 | -1.336e+03 | 0.0000 | 8156.0 | 41.30% | 7284.8 | 24.55% | motif file (matrix) | svg |
| 8 | A G T C C T G A A G T C C G A T C A G T G A T C A T G C A C T G A T C G G A C T | Fli1(ETS)/CD8-FLI-ChIP-Seq(GSE20898)/Homer | 1e-563 | -1.297e+03 | 0.0000 | 6653.0 | 33.69% | 5480.7 | 18.47% | motif file (matrix) | svg |
| 9 | T C G A T C G A T A G C G T A C T C A G T A C G C G T A C G T A T C A G A G C T | GABPA(ETS)/Jurkat-GABPa-ChIP-Seq(GSE17954)/Homer | 1e-551 | -1.270e+03 | 0.0000 | 5763.0 | 29.18% | 4473.5 | 15.08% | motif file (matrix) | svg |
| 10 | T C G A C T G A T A G C T G A C T C A G T C A G C G T A C G T A T C A G A G C T | ETV1(ETS)/GIST48-ETV1-ChIP-Seq(GSE22441)/Homer | 1e-531 | -1.223e+03 | 0.0000 | 7589.0 | 38.43% | 6751.5 | 22.76% | motif file (matrix) | svg |
| 11 | C G T A T A G C T A G C T G C A A C T G C T A G C G T A C G T A T C A G G A C T | EHF(ETS)/LoVo-EHF-ChIP-Seq(GSE49402)/Homer | 1e-527 | -1.216e+03 | 0.0000 | 6931.0 | 35.09% | 5943.2 | 20.03% | motif file (matrix) | svg |
| 12 | C G T A T G A C T A G C T G C A A C T G A C T G C G T A C G T A T C A G G A C T | ELF3(ETS)/PDAC-ELF3-ChIP-Seq(GSE64557)/Homer | 1e-513 | -1.181e+03 | 0.0000 | 4569.0 | 23.13% | 3257.8 | 10.98% | motif file (matrix) | svg |
| 13 | C T G A T A G C T G A C T C A G C T A G G T C A C G T A T C A G A G C T T C A G | ETV4(ETS)/HepG2-ETV4-ChIP-Seq(ENCODE)/Homer | 1e-431 | -9.928e+02 | 0.0000 | 6396.0 | 32.38% | 5655.9 | 19.06% | motif file (matrix) | svg |
| 14 | T G C A C T G A A T G C G T C A A C T G A C T G C G T A C G T A C T A G A G C T | Ets1-distal(ETS)/CD4+-PolII-ChIP-Seq(Barski\_et\_al.)/Homer | 1e-387 | -8.911e+02 | 0.0000 | 2229.0 | 11.29% | 1221.5 | 4.12% | motif file (matrix) | svg |
| 15 | C T G A T G C A T A G C T G A C T A C G T C A G C T G A G C T A T C A G G A C T | ELF1(ETS)/Jurkat-ELF1-ChIP-Seq(SRA014231)/Homer | 1e-363 | -8.366e+02 | 0.0000 | 3766.0 | 19.07% | 2826.4 | 9.53% | motif file (matrix) | svg |
| 16 | T C G A A G C T A C G T A C G T A G T C A G T C A C G T A T C G G A C T A T C G | EWS:ERG-fusion(ETS)/CADO\_ES1-EWS:ERG-ChIP-Seq(SRA014231)/Homer | 1e-331 | -7.633e+02 | 0.0000 | 3657.0 | 18.52% | 2808.5 | 9.47% | motif file (matrix) | svg |
| 17 | C T A G C T A G C G T A C G T A T A C G C G A T C T A G C T G A C T G A C G T A T A C G G A C T | PU.1:IRF8(ETS:IRF)/pDC-Irf8-ChIP-Seq(GSE66899)/Homer | 1e-331 | -7.623e+02 | 0.0000 | 1471.0 | 7.45% | 670.9 | 2.26% | motif file (matrix) | svg |
| 18 | T G C A T C G A T A G C G T A C T C A G C T A G G T C A G C T A T C A G G A C T | ETS(ETS)/Promoter/Homer | 1e-314 | -7.245e+02 | 0.0000 | 2648.0 | 13.41% | 1786.8 | 6.02% | motif file (matrix) | svg |
| 19 | T C A G T C A G G C T A C G T A T A C G G A C T T C A G T C G A C T G A C G T A T A C G G A C T | IRF8(IRF)/BMDM-IRF8-ChIP-Seq(GSE77884)/Homer | 1e-302 | -6.970e+02 | 0.0000 | 2058.0 | 10.42% | 1236.1 | 4.17% | motif file (matrix) | svg |
| 20 | T G A C C T A G T C A G G T C A C G T A T C A G C G A T T C A G T C G A T G C A C T G A T A G C | PU.1-IRF(ETS:IRF)/Bcell-PU.1-ChIP-Seq(GSE21512)/Homer | 1e-245 | -5.652e+02 | 0.0000 | 5817.0 | 29.45% | 5783.5 | 19.49% | motif file (matrix) | svg |
| 21 | T G C A C T G A A G T C G T C A A C T G A C T G C G T A C G T A C T G A A G C T | EWS:FLI1-fusion(ETS)/SK\_N\_MC-EWS:FLI1-ChIP-Seq(SRA014231)/Homer | 1e-239 | -5.518e+02 | 0.0000 | 3247.0 | 16.44% | 2672.6 | 9.01% | motif file (matrix) | svg |
| 22 | G A T C C T G A A G T C C G A T C G A T G A T C A G T C A C T G A T C G A G C T | Elk4(ETS)/Hela-Elk4-ChIP-Seq(GSE31477)/Homer | 1e-227 | -5.248e+02 | 0.0000 | 3510.0 | 17.77% | 3022.3 | 10.19% | motif file (matrix) | svg |
| 23 | G A T C T C G A A G T C C G A T C G A T A G T C A T G C A C T G A T C G G A C T | Elk1(ETS)/Hela-Elk1-ChIP-Seq(GSE31477)/Homer | 1e-219 | -5.046e+02 | 0.0000 | 3560.0 | 18.03% | 3118.6 | 10.51% | motif file (matrix) | svg |
| 24 | C T G A A T G C C G T A A C G T A G T C A G T C A C G T A C T G A T C G G C A T | SPDEF(ETS)/VCaP-SPDEF-ChIP-Seq(SRA014231)/Homer | 1e-185 | -4.270e+02 | 0.0000 | 4608.0 | 23.33% | 4571.9 | 15.41% | motif file (matrix) | svg |
| 25 | T G C A A G C T C T G A A T C G G A C T C T A G G T A C G A T C G T C A A G T C G T A C G A C T C T A G A T C G G C A T C A T G C A T G G A T C G T A C C T G A | CTCF(Zf)/CD4+-CTCF-ChIP-Seq(Barski\_et\_al.)/Homer | 1e-184 | -4.239e+02 | 0.0000 | 1124.0 | 5.69% | 627.2 | 2.11% | motif file (matrix) | svg |
| 26 | C T G A T A C G G C A T A G C T A G C T A G T C T C G A A C T G C A G T A G C T A G C T G A T C | IRF3(IRF)/BMDM-Irf3-ChIP-Seq(GSE67343)/Homer | 1e-154 | -3.553e+02 | 0.0000 | 1610.0 | 8.15% | 1177.3 | 3.97% | motif file (matrix) | svg |
| 27 | T A G C A G T C T G A C A G T C C T A G A T C G A G T C C A T G T G A C A G T C G T A C A G T C A G T C G C A T C T A G A T C G G C A T A C T G A T C G G A T C | BORIS(Zf)/K562-CTCFL-ChIP-Seq(GSE32465)/Homer | 1e-122 | -2.815e+02 | 0.0000 | 1473.0 | 7.46% | 1138.9 | 3.84% | motif file (matrix) | svg |
| 28 | T C G A T A G C G T C A A C T G C T A G C G T A C G A T A C T G A C G T A C T G A C T G A C G T | ETS:RUNX(ETS,Runt)/Jurkat-RUNX1-ChIP-Seq(GSE17954)/Homer | 1e-103 | -2.376e+02 | 0.0000 | 687.0 | 3.48% | 401.7 | 1.35% | motif file (matrix) | svg |
| 29 | C T A G T C G A C T G A C G T A T A C G G A C T T C A G T C G A G T C A T G C A T A C G A G C T | IRF2(IRF)/Erythroblas-IRF2-ChIP-Seq(GSE36985)/Homer | 1e-95 | -2.189e+02 | 0.0000 | 627.0 | 3.17% | 364.7 | 1.23% | motif file (matrix) | svg |
| 30 | T C A G C T G A C G T A C G T A T A C G G C A T C T A G C T G A C G T A C G T A T A C G G A C T | IRF1(IRF)/PBMC-IRF1-ChIP-Seq(GSE43036)/Homer | 1e-92 | -2.137e+02 | 0.0000 | 773.0 | 3.91% | 507.2 | 1.71% | motif file (matrix) | svg |
| 31 | C A T G A G T C A G C T C G T A C G A T C G A T G C A T G C A T C G A T C T G A C A T G T G A C | Mef2d(MADS)/Retina-Mef2d-ChIP-Seq(GSE61391)/Homer | 1e-69 | -1.598e+02 | 0.0000 | 657.0 | 3.33% | 457.2 | 1.54% | motif file (matrix) | svg |
| 32 | C A T G A G T C G A C T C G T A C G A T G C A T G A C T G C A T C G A T C T A G C A T G T G A C | Mef2b(MADS)/HEK293-Mef2b.V5-ChIP-Seq(GSE67450)/Homer | 1e-66 | -1.533e+02 | 0.0000 | 2295.0 | 11.62% | 2393.9 | 8.07% | motif file (matrix) | svg |
| 33 | A G T C A C G T A C T G A G C T A C G T A C G T G T C A A G T C | Foxo1(Forkhead)/RAW-Foxo1-ChIP-Seq(Fan\_et\_al.)/Homer | 1e-66 | -1.527e+02 | 0.0000 | 5983.0 | 30.29% | 7377.3 | 24.86% | motif file (matrix) | svg |
| 34 | C A T G G T A C G A C T G C T A C G T A C G T A C G T A G C T A G A C T C T G A T C A G G T A C | Mef2c(MADS)/GM12878-Mef2c-ChIP-Seq(GSE32465)/Homer | 1e-60 | -1.384e+02 | 0.0000 | 1258.0 | 6.37% | 1159.9 | 3.91% | motif file (matrix) | svg |
| 35 | T A C G C T A G A T G C G A T C G T A C A G T C C T A G A G T C A G T C A G T C G T A C A G T C | Sp1(Zf)/Promoter/Homer | 1e-55 | -1.288e+02 | 0.0000 | 2020.0 | 10.23% | 2122.9 | 7.16% | motif file (matrix) | svg |
| 36 | T C A G A C G T A G T C T C G A A G T C T C A G G C A T C T A G C T A G A G C T | Usf2(bHLH)/C2C12-Usf2-ChIP-Seq(GSE36030)/Homer | 1e-55 | -1.286e+02 | 0.0000 | 1243.0 | 6.29% | 1164.6 | 3.93% | motif file (matrix) | svg |
| 37 | G T A C G A C T C G T A C T G A T C G A C G T A G C T A C A G T C T G A T A C G | Mef2a(MADS)/HL1-Mef2a.biotin-ChIP-Seq(GSE21529)/Homer | 1e-53 | -1.229e+02 | 0.0000 | 1239.0 | 6.27% | 1173.6 | 3.96% | motif file (matrix) | svg |
| 38 | T C A G A G C T A T G C C G T A A G C T T C A G C A G T A C T G C T G A A G T C | MITF(bHLH)/MastCells-MITF-ChIP-Seq(GSE48085)/Homer | 1e-46 | -1.069e+02 | 0.0000 | 3089.0 | 15.64% | 3610.2 | 12.17% | motif file (matrix) | svg |
| 39 | T C A G A G C T A T G C C G T A A G T C T C A G A C G T A T C G T C G A A G T C G A T C T G A C | TFE3(bHLH)/MEF-TFE3-ChIP-Seq(GSE75757)/Homer | 1e-43 | -1.012e+02 | 0.0000 | 441.0 | 2.23% | 315.3 | 1.06% | motif file (matrix) | svg |
| 40 | C T G A A T C G A G C T A G C T A C G T T A G C C T G A T A C G C G A T A C G T G A C T A G T C | ISRE(IRF)/ThioMac-LPS-Expression(GSE23622)/Homer | 1e-39 | -9.207e+01 | 0.0000 | 330.0 | 1.67% | 216.9 | 0.73% | motif file (matrix) | svg |
| 41 | G A C T C T A G G A T C C A G T A C T G C T G A A T G C G C A T A T G C C T G A | MafA(bZIP)/Islet-MafA-ChIP-Seq(GSE30298)/Homer | 1e-39 | -9.186e+01 | 0.0000 | 2922.0 | 14.79% | 3456.3 | 11.65% | motif file (matrix) | svg |
| 42 | A C T G G A T C G A C T A C T G A C G T C A T G A C T G A C G T A G C T C G A T | RUNX-AML(Runt)/CD4+-PolII-ChIP-Seq(Barski\_et\_al.)/Homer | 1e-37 | -8.529e+01 | 0.0000 | 2340.0 | 11.85% | 2705.3 | 9.12% | motif file (matrix) | svg |
| 43 | C T G A T C A G C A G T C T A G A C T G C T A G G A T C A T C G A C T G C T G A T C A G G A T C | Sp5(Zf)/mES-Sp5.Flag-ChIP-Seq(GSE72989)/Homer | 1e-36 | -8.359e+01 | 0.0000 | 4805.0 | 24.33% | 6114.2 | 20.61% | motif file (matrix) | svg |
| 44 | T C G A A C T G A C T G C G T A C G T A T C G A A G T C C T G A A T C G G T A C G C A T C A T G | ETS:E-box(ETS,bHLH)/HPC7-Scl-ChIP-Seq(GSE22178)/Homer | 1e-36 | -8.311e+01 | 0.0000 | 419.0 | 2.12% | 318.4 | 1.07% | motif file (matrix) | svg |
| 45 | T A G C G C T A T C G A C T G A A G T C A G T C C T G A A G T C C G T A C T A G | RUNX(Runt)/HPC7-Runx1-ChIP-Seq(GSE22178)/Homer | 1e-35 | -8.261e+01 | 0.0000 | 2466.0 | 12.49% | 2886.2 | 9.73% | motif file (matrix) | svg |
| 46 | A G T C C T A G C T A G A G T C G A T C G T A C A G T C C T A G A G T C A G T C A G T C G T A C | Sp2(Zf)/HEK293-Sp2.eGFP-ChIP-Seq(Encode)/Homer | 1e-35 | -8.230e+01 | 0.0000 | 6328.0 | 32.04% | 8299.9 | 27.97% | motif file (matrix) | svg |
| 47 | G A C T T C A G C T A G A G T C A G T C G T A C A G T C C T G A A G T C A G T C A G T C G A C T A G T C A C T G A T G C | KLF3(Zf)/MEF-Klf3-ChIP-Seq(GSE44748)/Homer | 1e-35 | -8.204e+01 | 0.0000 | 2614.0 | 13.24% | 3089.0 | 10.41% | motif file (matrix) | svg |
| 48 | A T G C T C A G T C G A G C A T A C T G C G T A A G T C T C A G G A C T T G A C C G T A A G C T | Atf2(bZIP)/3T3L1-Atf2-ChIP-Seq(GSE56872)/Homer | 1e-35 | -8.062e+01 | 0.0000 | 1044.0 | 5.29% | 1049.5 | 3.54% | motif file (matrix) | svg |
| 49 | T C G A G C A T A C T G C T G A A G T C T C A G G A C T G T A C C G T A A G C T A G T C G A T C | c-Jun-CRE(bZIP)/K562-cJun-ChIP-Seq(GSE31477)/Homer | 1e-34 | -7.883e+01 | 0.0000 | 932.0 | 4.72% | 917.9 | 3.09% | motif file (matrix) | svg |
| 50 | G C T A C T G A T C G A A G T C A G T C C T G A A G T C G T C A C T G A T G C A | RUNX1(Runt)/Jurkat-RUNX1-ChIP-Seq(GSE29180)/Homer | 1e-34 | -7.869e+01 | 0.0000 | 3390.0 | 17.16% | 4166.7 | 14.04% | motif file (matrix) | svg |
| 51 | T G C A A G C T A C G T C T A G G A T C C T A G G A T C G T C A C T G A A G T C | CEBP(bZIP)/ThioMac-CEBPb-ChIP-Seq(GSE21512)/Homer | 1e-33 | -7.743e+01 | 0.0000 | 1673.0 | 8.47% | 1859.4 | 6.27% | motif file (matrix) | svg |
| 52 | G C T A T A G C A G C T A T C G G T C A C G T A G C T A A T G C G A T C C T G A | IRF4(IRF)/GM12878-IRF4-ChIP-Seq(GSE32465)/Homer | 1e-32 | -7.580e+01 | 0.0000 | 1472.0 | 7.45% | 1605.6 | 5.41% | motif file (matrix) | svg |
| 53 | T C A G C T A G C T A G A C T G A C T G G A T C A C T G A C T G C T A G C T A G A G T C G A T C | KLF1(Zf)/HUDEP2-KLF1-CutnRun(GSE136251)/Homer | 1e-32 | -7.540e+01 | 0.0000 | 4254.0 | 21.54% | 5391.7 | 18.17% | motif file (matrix) | svg |
| 54 | T C G A A C G T A C G T C T G A G A T C T C A G G A C T G T C A C G T A A G C T G T C A C T A G A G C T A C G T T C G A | NFIL3(bZIP)/HepG2-NFIL3-ChIP-Seq(Encode)/Homer | 1e-31 | -7.308e+01 | 0.0000 | 1589.0 | 8.05% | 1767.9 | 5.96% | motif file (matrix) | svg |
| 55 | T G C A T C G A T C G A A C G T C A T G C G T A A G T C T C A G A C G T G T A C C G T A A G C T | CREB5(bZIP)/LNCaP-CREB5.V5-ChIP-Seq(GSE137775)/Homer | 1e-31 | -7.307e+01 | 0.0000 | 1037.0 | 5.25% | 1063.4 | 3.58% | motif file (matrix) | svg |
| 56 | T A G C C T A G T C G A G A C T A C T G C T G A A G T C T C A G G C A T T G A C C T G A A G C T | Atf7(bZIP)/3T3L1-Atf7-ChIP-Seq(GSE56872)/Homer | 1e-31 | -7.256e+01 | 0.0000 | 1395.0 | 7.06% | 1518.5 | 5.12% | motif file (matrix) | svg |
| 57 | T A C G T C A G A G C T A T G C C G T A A G T C T C A G A C G T A C T G T C G A | USF1(bHLH)/GM12878-Usf1-ChIP-Seq(GSE32465)/Homer | 1e-30 | -7.090e+01 | 0.0000 | 1632.0 | 8.26% | 1832.3 | 6.18% | motif file (matrix) | svg |
| 58 | A T G C A T G C A T C G T A C G A G C T A G T C G C T A A G T C T C A G G A C T A C T G T C G A | E-box(bHLH)/Promoter/Homer | 1e-30 | -7.035e+01 | 0.0000 | 424.0 | 2.15% | 344.4 | 1.16% | motif file (matrix) | svg |
| 59 | A G T C G A C T C A G T A C T G C T A G T G A C G C T A A T G C G C A T A T C G C G A T A C T G G A T C G T A C G T C A C T G A | NF1(CTF)/LNCAP-NF1-ChIP-Seq(Unpublished)/Homer | 1e-29 | -6.739e+01 | 0.0000 | 1396.0 | 7.07% | 1539.7 | 5.19% | motif file (matrix) | svg |
| 60 | A T G C A T C G T A C G A G C T A T C G C T G A A G T C C T A G A G C T A T G C C T G A A T G C | CRE(bZIP)/Promoter/Homer | 1e-29 | -6.704e+01 | 0.0000 | 923.0 | 4.67% | 940.9 | 3.17% | motif file (matrix) | svg |
| 61 | T A C G T C G A G A C T A C T G C T G A A G T C T C A G G A C T T G A C C T G A | Atf1(bZIP)/K562-ATF1-ChIP-Seq(GSE31477)/Homer | 1e-27 | -6.323e+01 | 0.0000 | 1933.0 | 9.79% | 2265.9 | 7.64% | motif file (matrix) | svg |
| 62 | G T A C C A G T A C T G A C T G A C T G G A T C A C T G A C G T A C T G A C T G A G T C G A T C | KLF6(Zf)/PDAC-KLF6-ChIP-Seq(GSE64557)/Homer | 1e-27 | -6.284e+01 | 0.0000 | 4505.0 | 22.81% | 5834.3 | 19.66% | motif file (matrix) | svg |
| 63 | C A T G C T A G T C G A A C G T A C T G C G T A T A G C C G A T T G A C C G T A A G C T G A T C | Fra2(bZIP)/Striatum-Fra2-ChIP-Seq(GSE43429)/Homer | 1e-27 | -6.256e+01 | 0.0000 | 1335.0 | 6.76% | 1479.6 | 4.99% | motif file (matrix) | svg |
| 64 | A G C T G C A T A C T G A C G T A G T C A C G T C T A G T A C G | Smad3(MAD)/NPC-Smad3-ChIP-Seq(GSE36673)/Homer | 1e-26 | -6.147e+01 | 0.0000 | 9010.0 | 45.62% | 12407.3 | 41.82% | motif file (matrix) | svg |
| 65 | C G A T T A C G T G A C G A C T C A T G C G T A T A C G A C G T G T A C C T G A | Bach2(bZIP)/OCILy7-Bach2-ChIP-Seq(GSE44420)/Homer | 1e-25 | -5.974e+01 | 0.0000 | 673.0 | 3.41% | 655.5 | 2.21% | motif file (matrix) | svg |
| 66 | A C T G C A T G T C G A A C G T A C T G C G T A A T C G A C G T G T A C C G T A G A C T A G T C | Fos(bZIP)/TSC-Fos-ChIP-Seq(GSE110950)/Homer | 1e-25 | -5.932e+01 | 0.0000 | 1556.0 | 7.88% | 1782.5 | 6.01% | motif file (matrix) | svg |
| 67 | T C G A A C G T A C T G C T G A A G T C T C A G A G C T G T A C C G T A A G C T G A T C T C G A | JunD(bZIP)/K562-JunD-ChIP-Seq/Homer | 1e-25 | -5.915e+01 | 0.0000 | 354.0 | 1.79% | 287.1 | 0.97% | motif file (matrix) | svg |
| 68 | A C T G C T A G T C G A C G A T C A T G G C T A A T C G C G A T G T A C G C T A A G C T G T A C | Fra1(bZIP)/BT549-Fra1-ChIP-Seq(GSE46166)/Homer | 1e-25 | -5.892e+01 | 0.0000 | 1478.0 | 7.48% | 1681.2 | 5.67% | motif file (matrix) | svg |
| 69 | C G T A G A C T C G A T A T C G G T A C G C A T C A T G C G T A T A C G G C A T G T A C C G T A C A T G A T G C G C T A C T A G G C A T G C A T G C A T G A C T | MafB(bZIP)/BMM-Mafb-ChIP-Seq(GSE75722)/Homer | 1e-23 | -5.304e+01 | 0.0000 | 1408.0 | 7.13% | 1615.1 | 5.44% | motif file (matrix) | svg |
| 70 | G T A C C G T A C G T A T A C G G C A T G T A C C G T A C A T G A G T C C G T A C G T A C G A T G C A T G C A T G A C T | MafF(bZIP)/HepG2-MafF-ChIP-Seq(GSE31477)/Homer | 1e-22 | -5.257e+01 | 0.0000 | 737.0 | 3.73% | 754.9 | 2.54% | motif file (matrix) | svg |
| 71 | A G T C C G A T A C T G A T C G T G A C G C T A C A T G A T C G T G A C C G A T A C T G T A G C G T A C G T C A | Tlx?(NR)/NPC-H3K4me1-ChIP-Seq(GSE16256)/Homer | 1e-22 | -5.180e+01 | 0.0000 | 1504.0 | 7.62% | 1748.7 | 5.89% | motif file (matrix) | svg |
| 72 | A G C T A G T C A G T C A C G T C T A G A C G T A C G T A C G T C G T A A G T C G A T C C G T A | FOXP1(Forkhead)/H9-FOXP1-ChIP-Seq(GSE31006)/Homer | 1e-22 | -5.082e+01 | 0.0000 | 1185.0 | 6.00% | 1332.2 | 4.49% | motif file (matrix) | svg |
| 73 | C T A G T C G A A C G T A C T G C G T A A T G C A C G T G T A C C G T A A G C T G A T C G T A C | Atf3(bZIP)/GBM-ATF3-ChIP-Seq(GSE33912)/Homer | 1e-21 | -5.054e+01 | 0.0000 | 1751.0 | 8.87% | 2085.1 | 7.03% | motif file (matrix) | svg |
| 74 | T C A G T G A C G T A C T G C A G T A C C T A G G T A C A T G C A G T C G T C A A G T C G A C T | Klf9(Zf)/GBM-Klf9-ChIP-Seq(GSE62211)/Homer | 1e-21 | -5.016e+01 | 0.0000 | 2024.0 | 10.25% | 2457.3 | 8.28% | motif file (matrix) | svg |
| 75 | A T G C T C G A A G T C A G C T A C G T G T A C A G T C G C T A C T A G C A T G G T C A C T G A T C A G A G T C | Stat3+il21(Stat)/CD4-Stat3-ChIP-Seq(GSE19198)/Homer | 1e-21 | -4.918e+01 | 0.0000 | 2330.0 | 11.80% | 2882.5 | 9.72% | motif file (matrix) | svg |
| 76 | C T A G T C G A C G A T A C T G C G T A T A C G A G C T T G A C G C T A A C G T G A T C T A G C | Fosl2(bZIP)/3T3L1-Fosl2-ChIP-Seq(GSE56872)/Homer | 1e-21 | -4.862e+01 | 0.0000 | 976.0 | 4.94% | 1070.6 | 3.61% | motif file (matrix) | svg |
| 77 | T G A C G C T A T C G A T G C A A G T C A G T C C G T A A G T C C G T A C T G A G C T A G T A C | RUNX2(Runt)/PCa-RUNX2-ChIP-Seq(GSE33889)/Homer | 1e-21 | -4.851e+01 | 0.0000 | 2671.0 | 13.52% | 3358.0 | 11.32% | motif file (matrix) | svg |
| 78 | C T A G T C G A G C A T C A T G G C T A T A G C C G A T G T A C C T G A A G C T | JunB(bZIP)/DendriticCells-Junb-ChIP-Seq(GSE36099)/Homer | 1e-20 | -4.737e+01 | 0.0000 | 1472.0 | 7.45% | 1727.1 | 5.82% | motif file (matrix) | svg |
| 79 | G A C T A G T C C G A T A C T G C T G A T G A C G T A C C G T A A T C G G C A T C T G A C T A G | Bcl11a(Zf)/HSPC-BCL11A-ChIP-Seq(GSE104676)/Homer | 1e-20 | -4.651e+01 | 0.0000 | 2187.0 | 11.07% | 2702.1 | 9.11% | motif file (matrix) | svg |
| 80 | C G A T C T A G A C G T G T C A C G T A C G T A A G T C C G T A | Foxo3(Forkhead)/U2OS-Foxo3-ChIP-Seq(E-MTAB-2701)/Homer | 1e-20 | -4.631e+01 | 0.0000 | 1874.0 | 9.49% | 2275.5 | 7.67% | motif file (matrix) | svg |
| 81 | T C G A A C G T C A T G G C T A T A G C C G A T G T A C G C T A A C G T A T G C | AP-1(bZIP)/ThioMac-PU.1-ChIP-Seq(GSE21512)/Homer | 1e-19 | -4.587e+01 | 0.0000 | 1955.0 | 9.90% | 2388.4 | 8.05% | motif file (matrix) | svg |
| 82 | C A G T T G C A A C G T A C T G C G T A A T C G C G A T T G A C C G T A A C G T | BATF(bZIP)/Th17-BATF-ChIP-Seq(GSE39756)/Homer | 1e-19 | -4.512e+01 | 0.0000 | 1738.0 | 8.80% | 2097.8 | 7.07% | motif file (matrix) | svg |
| 83 | C T G A T C A G G T A C G C T A A C T G T G A C G C A T C A T G | SCL(bHLH)/HPC7-Scl-ChIP-Seq(GSE13511)/Homer | 1e-19 | -4.448e+01 | 0.0000 | 12630.0 | 63.95% | 18040.4 | 60.80% | motif file (matrix) | svg |
| 84 | C T A G G C A T G A T C C G T A A G T C T C A G G A C T C T A G | CLOCK(bHLH)/Liver-Clock-ChIP-Seq(GSE39860)/Homer | 1e-19 | -4.414e+01 | 0.0000 | 1850.0 | 9.37% | 2255.6 | 7.60% | motif file (matrix) | svg |
| 85 | A G C T A C G T A C T G A T G C A G T C C G T A C T G A T A C G | NF1-halfsite(CTF)/LNCaP-NF1-ChIP-Seq(Unpublished)/Homer | 1e-19 | -4.388e+01 | 0.0000 | 5384.0 | 27.26% | 7258.4 | 24.46% | motif file (matrix) | svg |
| 86 | T C G A T G A C G C A T A G C T C A G T G A T C G C T A G A T C G A C T A C G T G C A T A G T C | PRDM1(Zf)/Hela-PRDM1-ChIP-Seq(GSE31477)/Homer | 1e-19 | -4.383e+01 | 0.0000 | 1812.0 | 9.17% | 2205.4 | 7.43% | motif file (matrix) | svg |
| 87 | C T A G T C G A A C G T A C T G C G T A T A G C C G A T G T A C C G T A A G C T G A T C G T A C | Jun-AP1(bZIP)/K562-cJun-ChIP-Seq(GSE31477)/Homer | 1e-18 | -4.245e+01 | 0.0000 | 731.0 | 3.70% | 780.9 | 2.63% | motif file (matrix) | svg |
| 88 | T C A G G A C T C A G T C T G A A G C T C T A G G A C T T G C A C T G A A G T C | HLF(bZIP)/HSC-HLF.Flag-ChIP-Seq(GSE69817)/Homer | 1e-17 | -4.142e+01 | 0.0000 | 1868.0 | 9.46% | 2296.3 | 7.74% | motif file (matrix) | svg |
| 89 | C G T A C T A G A C T G A C T G G A C T C T A G C A G T C T A G C A T G G A T C | KLF5(Zf)/LoVo-KLF5-ChIP-Seq(GSE49402)/Homer | 1e-17 | -4.089e+01 | 0.0000 | 5373.0 | 27.21% | 7272.7 | 24.51% | motif file (matrix) | svg |
| 90 | T G C A T A G C G A C T T G C A T G A C T G C A C G T A A G C T A G C T A G T C A G T C G T A C | GFY(?)/Promoter/Homer | 1e-16 | -3.906e+01 | 0.0000 | 340.0 | 1.72% | 311.8 | 1.05% | motif file (matrix) | svg |
| 91 | T A C G T C G A C A G T A C T G G C T A A T G C C G A T G T A C C G T A A C T G T A G C C G T A | NF-E2(bZIP)/K562-NFE2-ChIP-Seq(GSE31477)/Homer | 1e-16 | -3.858e+01 | 0.0000 | 225.0 | 1.14% | 183.0 | 0.62% | motif file (matrix) | svg |
| 92 | C T A G G T A C A G T C T G C A A G T C C T G A A G T C A G T C A G T C G C T A | Klf4(Zf)/mES-Klf4-ChIP-Seq(GSE11431)/Homer | 1e-16 | -3.789e+01 | 0.0000 | 1651.0 | 8.36% | 2021.2 | 6.81% | motif file (matrix) | svg |
| 93 | T C A G A G C T A C G T A C G T G T A C G A T C C G T A C T A G C A T G G T C A C G T A T C G A | STAT4(Stat)/CD4-Stat4-ChIP-Seq(GSE22104)/Homer | 1e-16 | -3.745e+01 | 0.0000 | 2798.0 | 14.17% | 3617.5 | 12.19% | motif file (matrix) | svg |
| 94 | T A C G T A G C G C T A C G A T C T A G A C G T C A G T C A G T G C T A A G T C G T C A G C A T | FOXK2(Forkhead)/U2OS-FOXK2-ChIP-Seq(E-MTAB-2204)/Homer | 1e-16 | -3.725e+01 | 0.0000 | 1684.0 | 8.53% | 2070.1 | 6.98% | motif file (matrix) | svg |
| 95 | T C A G C G T A A G T C A G C T C G T A A G T C C T G A C G T A A G T C G C A T A G T C A G T C A G T C C T G A A C T G T G C A T C G A C A T G A T C G G A T C | Ronin(THAP)/ES-Thap11-ChIP-Seq(GSE51522)/Homer | 1e-16 | -3.696e+01 | 0.0000 | 216.0 | 1.09% | 175.5 | 0.59% | motif file (matrix) | svg |
| 96 | A G C T T G A C C G A T C G A T C T A G A C G T C A G T C A G T G C T A A G T C | FOXK1(Forkhead)/HEK293-FOXK1-ChIP-Seq(GSE51673)/Homer | 1e-15 | -3.642e+01 | 0.0000 | 2588.0 | 13.10% | 3330.4 | 11.22% | motif file (matrix) | svg |
| 97 | T A C G A T C G T A G C G A T C A C T G A C G T A G T C A C G T C T A G A T C G | Smad4(MAD)/ESC-SMAD4-ChIP-Seq(GSE29422)/Homer | 1e-15 | -3.576e+01 | 0.0000 | 5535.0 | 28.03% | 7562.5 | 25.49% | motif file (matrix) | svg |
| 98 | A T C G A G C T A C T G A G T C A C T G A G T C C G T A A C G T A C T G A G T C A C T G A G T C | NRF(NRF)/Promoter/Homer | 1e-15 | -3.566e+01 | 0.0000 | 966.0 | 4.89% | 1112.3 | 3.75% | motif file (matrix) | svg |
| 99 | G T C A G C A T A C T G G T A C G A C T A C T G G C T A A T C G C A G T G T A C C G T A A G C T | Nrf2(bZIP)/Lymphoblast-Nrf2-ChIP-Seq(GSE37589)/Homer | 1e-15 | -3.519e+01 | 0.0000 | 192.0 | 0.97% | 152.1 | 0.51% | motif file (matrix) | svg |
| 100 | C G T A C G T A C G T A G C A T G C A T T A C G G T A C G A C T A C T G C G T A A T C G A C G T G T A C C G T A A G C T | Bach1(bZIP)/K562-Bach1-ChIP-Seq(GSE31477)/Homer | 1e-15 | -3.501e+01 | 0.0000 | 188.0 | 0.95% | 148.1 | 0.50% | motif file (matrix) | svg |
| 101 | A T G C G A C T A C G T C T A G A C G T A C G T A C G T C T G A G A T C G C T A A G C T C G T A | Foxa2(Forkhead)/Liver-Foxa2-ChIP-Seq(GSE25694)/Homer | 1e-15 | -3.457e+01 | 0.0000 | 2051.0 | 10.38% | 2594.0 | 8.74% | motif file (matrix) | svg |
| 102 | A G C T C A T G G C A T G A T C T G C A C T A G G A T C A C G T | Tgif2(Homeobox)/mES-Tgif2-ChIP-Seq(GSE55404)/Homer | 1e-14 | -3.266e+01 | 0.0000 | 8722.0 | 44.16% | 12298.3 | 41.45% | motif file (matrix) | svg |
| 103 | T C A G T A G C G A C T C A T G C T G A A T C G G C A T G T A C C G T A A C T G T A G C T G C A | MafK(bZIP)/C2C12-MafK-ChIP-Seq(GSE36030)/Homer | 1e-14 | -3.240e+01 | 0.0000 | 852.0 | 4.31% | 977.5 | 3.29% | motif file (matrix) | svg |
| 104 | C A T G G T A C C G T A A G T C C T A G A C G T A C T G G T A C A G T C A G C T | bHLHE40(bHLH)/HepG2-BHLHE40-ChIP-Seq(GSE31477)/Homer | 1e-13 | -3.164e+01 | 0.0000 | 1063.0 | 5.38% | 1261.2 | 4.25% | motif file (matrix) | svg |
| 105 | T G C A A T G C A C G T A C G T A C G T A T G C C T A G A C G T A C G T A G C T G A T C A G C T | T1ISRE(IRF)/ThioMac-Ifnb-Expression/Homer | 1e-13 | -3.066e+01 | 0.0000 | 76.0 | 0.38% | 42.1 | 0.14% | motif file (matrix) | svg |
| 106 | A T G C C T G A G A C T A C G T A C G T G T A C G A T C C G A T C T A G C A T G C G T A C G T A C T G A G A C T | STAT1(Stat)/HelaS3-STAT1-ChIP-Seq(GSE12782)/Homer | 1e-12 | -2.797e+01 | 0.0000 | 884.0 | 4.48% | 1041.5 | 3.51% | motif file (matrix) | svg |
| 107 | G C A T G C A T C T G A A C G T C T G A A C G T C G T A C G T A C G T A A G T C G T C A G T C A | Foxf1(Forkhead)/Lung-Foxf1-ChIP-Seq(GSE77951)/Homer | 1e-12 | -2.791e+01 | 0.0000 | 2207.0 | 11.17% | 2864.6 | 9.66% | motif file (matrix) | svg |
| 108 | C T A G A C T G C T A G T C A G T C A G T A C G C T A G A C T G | Maz(Zf)/HepG2-Maz-ChIP-Seq(GSE31477)/Homer | 1e-12 | -2.790e+01 | 0.0000 | 5509.0 | 27.89% | 7617.3 | 25.67% | motif file (matrix) | svg |
| 109 | C T A G T C A G C A G T T C A G A C T G A C T G G A T C C T A G A C T G C T A G T C A G A T G C | KLF14(Zf)/HEK293-KLF14.GFP-ChIP-Seq(GSE58341)/Homer | 1e-11 | -2.727e+01 | 0.0000 | 6962.0 | 35.25% | 9761.8 | 32.90% | motif file (matrix) | svg |
| 110 | A G T C G C A T C G T A C G T A G T A C A C G T A C T G G A T C G A T C T C G A | BMYB(HTH)/Hela-BMYB-ChIP-Seq(GSE27030)/Homer | 1e-11 | -2.710e+01 | 0.0000 | 4410.0 | 22.33% | 6026.0 | 20.31% | motif file (matrix) | svg |
| 111 | A G T C G A C T C A G T G T A C A G T C A T C G T C A G A C T G G T C A C G T A | Stat3(Stat)/mES-Stat3-ChIP-Seq(GSE11431)/Homer | 1e-11 | -2.642e+01 | 0.0000 | 1681.0 | 8.51% | 2140.9 | 7.22% | motif file (matrix) | svg |
| 112 | C T G A A C G T A C G T A C G T A G T C G A C T C G A T C T G A A C T G C G T A C G T A T C G A | STAT5(Stat)/mCD4+-Stat5-ChIP-Seq(GSE12346)/Homer | 1e-11 | -2.573e+01 | 0.0000 | 1014.0 | 5.13% | 1228.7 | 4.14% | motif file (matrix) | svg |
| 113 | A T G C G A C T A C T G C A G T G A T C A C G T T A C G T A C G | Smad2(MAD)/ES-SMAD2-ChIP-Seq(GSE29422)/Homer | 1e-11 | -2.563e+01 | 0.0000 | 5357.0 | 27.12% | 7424.9 | 25.03% | motif file (matrix) | svg |
| 114 | A T G C A G C T T C A G T G A C T C A G A T G C T G C A A C G T A T C G G A T C A C T G A G T C | NRF1(NRF)/MCF7-NRF1-ChIP-Seq(Unpublished)/Homer | 1e-10 | -2.501e+01 | 0.0000 | 867.0 | 4.39% | 1034.2 | 3.49% | motif file (matrix) | svg |
| 115 | T C G A C T A G A G T C A G T C C G T A C G T A A C G T T A G C T C A G T A C G | NFY(CCAAT)/Promoter/Homer | 1e-9 | -2.289e+01 | 0.0000 | 2547.0 | 12.90% | 3392.1 | 11.43% | motif file (matrix) | svg |
| 116 | T G A C C T G A C T A G C T G A C G T A A G T C C T G A A C G T G C A T T A G C G C A T A T C G G A C T G A C T G A T C | GRE(NR),IR3/RAW264.7-GRE-ChIP-Seq(Unpublished)/Homer | 1e-9 | -2.188e+01 | 0.0000 | 696.0 | 3.52% | 822.5 | 2.77% | motif file (matrix) | svg |
| 117 | C T G A A G C T A C G T A C G T A G T C G A C T G A C T C T G A C T G A C T A G C G T A C G T A | STAT6(Stat)/CD4-Stat6-ChIP-Seq(GSE22104)/Homer | 1e-9 | -2.105e+01 | 0.0000 | 1414.0 | 7.16% | 1810.9 | 6.10% | motif file (matrix) | svg |
| 118 | G A C T G C A T C T A G C G A T G A T C T C G A C A T G G A T C | Tgif1(Homeobox)/mES-Tgif1-ChIP-Seq(GSE55404)/Homer | 1e-9 | -2.092e+01 | 0.0000 | 8125.0 | 41.14% | 11582.1 | 39.04% | motif file (matrix) | svg |
| 119 | A T G C A G T C G C A T A G C T A C G T T C A G C G A T A G C T G A T C A T C G | Sox10(HMG)/SciaticNerve-Sox3-ChIP-Seq(GSE35132)/Homer | 1e-9 | -2.081e+01 | 0.0000 | 4467.0 | 22.62% | 6189.0 | 20.86% | motif file (matrix) | svg |
| 120 | T G C A A G C T C A T G C G T A A G C T A C T G G A T C G T C A C G T A A G C T | Atf4(bZIP)/MEF-Atf4-ChIP-Seq(GSE35681)/Homer | 1e-8 | -2.037e+01 | 0.0000 | 603.0 | 3.05% | 706.6 | 2.38% | motif file (matrix) | svg |
| 121 | T C A G A C G T T C G A T A G C A G T C C G T A A C T G G T A C A C G T A C T G A T C G A G T C | Atoh1(bHLH)/Cerebellum-Atoh1-ChIP-Seq(GSE22111)/Homer | 1e-8 | -1.981e+01 | 0.0000 | 3341.0 | 16.92% | 4567.0 | 15.39% | motif file (matrix) | svg |
| 122 | G A C T C A G T G A T C G A T C A C G T G A T C C T G A T A C G C G T A G T C A | STAT6(Stat)/Macrophage-Stat6-ChIP-Seq(GSE38377)/Homer | 1e-8 | -1.956e+01 | 0.0000 | 1484.0 | 7.51% | 1921.0 | 6.47% | motif file (matrix) | svg |
| 123 | C T A G T C G A C G A T C T A G G C A T C A G T C T A G G A T C C G T A G T C A | CEBP:AP1(bZIP)/ThioMac-CEBPb-ChIP-Seq(GSE21512)/Homer | 1e-8 | -1.955e+01 | 0.0000 | 1779.0 | 9.01% | 2335.2 | 7.87% | motif file (matrix) | svg |
| 124 | T A G C C G T A C T G A T A C G C G T A A C G T A C T G A C T G A G T C T A C G C T A G G T A C | YY1(Zf)/Promoter/Homer | 1e-8 | -1.954e+01 | 0.0000 | 394.0 | 1.99% | 436.6 | 1.47% | motif file (matrix) | svg |
| 125 | G T C A T G C A G C T A A G T C C G T A A C T G T G A C G C A T T C A G C A G T | Ap4(bHLH)/AML-Tfap4-ChIP-Seq(GSE45738)/Homer | 1e-8 | -1.936e+01 | 0.0000 | 3829.0 | 19.39% | 5280.4 | 17.80% | motif file (matrix) | svg |
| 126 | A T G C C T G A A T C G T A C G A G T C C G A T T C A G C G A T C T A G A G C T G T C A G T C A C G T A A G T C C G T A T A C G C T G A | Fox:Ebox(Forkhead,bHLH)/Panc1-Foxa2-ChIP-Seq(GSE47459)/Homer | 1e-8 | -1.912e+01 | 0.0000 | 2737.0 | 13.86% | 3706.0 | 12.49% | motif file (matrix) | svg |
| 127 | C T A G A T G C A T G C C G A T A C T G G A C T A T G C G C T A T G A C A G C T T A G C G C T A | PBX1(Homeobox)/MCF7-PBX1-ChIP-Seq(GSE28007)/Homer | 1e-8 | -1.892e+01 | 0.0000 | 317.0 | 1.61% | 340.5 | 1.15% | motif file (matrix) | svg |
| 128 | T A C G T A C G G T A C A T C G T A C G T A C G G T C A C T G A C G T A G A C T | E2F4(E2F)/K562-E2F4-ChIP-Seq(GSE31477)/Homer | 1e-8 | -1.880e+01 | 0.0000 | 1774.0 | 8.98% | 2335.9 | 7.87% | motif file (matrix) | svg |
| 129 | G T A C C T A G T C A G A G C T T A G C C G T A A T G C T A C G A G T C G T A C G T C A A G T C | Srebp2(bHLH)/HepG2-Srebp2-ChIP-Seq(GSE31477)/Homer | 1e-8 | -1.861e+01 | 0.0000 | 398.0 | 2.02% | 445.7 | 1.50% | motif file (matrix) | svg |
| 130 | A T G C G A T C C G A T A C G T A C G T A C T G C A G T A G C T | Sox3(HMG)/NPC-Sox3-ChIP-Seq(GSE33059)/Homer | 1e-8 | -1.861e+01 | 0.0000 | 4631.0 | 23.45% | 6460.5 | 21.78% | motif file (matrix) | svg |
| 131 | G A C T C T A G C T A G A G T C T G C A A C T G A C G T A C G T C T A G T C A G | AMYB(HTH)/Testes-AMYB-ChIP-Seq(GSE44588)/Homer | 1e-7 | -1.828e+01 | 0.0000 | 4575.0 | 23.16% | 6383.8 | 21.52% | motif file (matrix) | svg |
| 132 | C G A T T A C G G A T C G C T A T C A G G A C T C G A T G T A C G A T C G T C A T C G A T G A C C G T A C T A G G A C T C T A G C T A G G T A C A G T C C G T A | CTCF-SatelliteElement(Zf?)/CD4+-CTCF-ChIP-Seq(Barski\_et\_al.)/Homer | 1e-7 | -1.827e+01 | 0.0000 | 88.0 | 0.45% | 68.3 | 0.23% | motif file (matrix) | svg |
| 133 | C G A T T G C A T G C A G A T C C G T A A C T G T G A C G A C T C A T G A C T G | Tcf21(bHLH)/ArterySmoothMuscle-Tcf21-ChIP-Seq(GSE61369)/Homer | 1e-7 | -1.821e+01 | 0.0000 | 2992.0 | 15.15% | 4083.2 | 13.76% | motif file (matrix) | svg |
| 134 | T C A G T A C G T A G C A C G T A C T G C G A T A G T C C G T A T A C G A G T C | Meis1(Homeobox)/MastCells-Meis1-ChIP-Seq(GSE48085)/Homer | 1e-7 | -1.815e+01 | 0.0000 | 5138.0 | 26.02% | 7210.4 | 24.30% | motif file (matrix) | svg |
| 135 | T C G A T A G C T G A C C T G A A G T C A C T G G A C T C A T G | c-Myc(bHLH)/LNCAP-cMyc-ChIP-Seq(Unpublished)/Homer | 1e-7 | -1.760e+01 | 0.0000 | 1652.0 | 8.36% | 2174.3 | 7.33% | motif file (matrix) | svg |
| 136 | T A G C T A G C G A C T C T A G A G C T A G T C G T C A T G C A A C G T A T G C G C T A T G C A | Pbx3(Homeobox)/GM12878-PBX3-ChIP-Seq(GSE32465)/Homer | 1e-7 | -1.746e+01 | 0.0000 | 832.0 | 4.21% | 1032.7 | 3.48% | motif file (matrix) | svg |
| 137 | C G T A C G T A C G T A G C A T G C A T A C T G G T A C G A C T C T A G G C T A T A C G G A C T T G A C C G T A A G C T | NFE2L2(bZIP)/HepG2-NFE2L2-ChIP-Seq(Encode)/Homer | 1e-7 | -1.720e+01 | 0.0000 | 182.0 | 0.92% | 178.0 | 0.60% | motif file (matrix) | svg |
| 138 | C A T G A C T G A G C T A T G C C G T A A T G C G T A C G A C T T A C G C T G A A C T G A C T G G C A T A T G C C T G A | THRb(NR)/HepG2-THRb.Flag-ChIP-Seq(Encode)/Homer | 1e-7 | -1.692e+01 | 0.0000 | 1830.0 | 9.27% | 2433.3 | 8.20% | motif file (matrix) | svg |
| 139 | A T G C G A C T A G C T A G C T A G T C G C T A C A G T C G A T G C T A A C G T A C T G G C T A T A G C G C A T T G A C | IRF:BATF(IRF:bZIP)/pDC-Irf8-ChIP-Seq(GSE66899)/Homer | 1e-7 | -1.691e+01 | 0.0000 | 350.0 | 1.77% | 390.5 | 1.32% | motif file (matrix) | svg |
| 140 | T A C G A T G C G A C T A C T G A G C T A G T C G T C A T G C A A C G T A G T C G C T A T G C A | Pknox1(Homeobox)/ES-Prep1-ChIP-Seq(GSE63282)/Homer | 1e-7 | -1.687e+01 | 0.0000 | 786.0 | 3.98% | 973.2 | 3.28% | motif file (matrix) | svg |
| 141 | C G T A C A T G C A T G A C T G C T A G T C G A G C A T C G A T A G C T A G T C G A T C G T A C | NFkB-p65(RHD)/GM12787-p65-ChIP-Seq(GSE19485)/Homer | 1e-7 | -1.627e+01 | 0.0000 | 1434.0 | 7.26% | 1879.9 | 6.34% | motif file (matrix) | svg |
| 142 | A C T G C A T G G C T A T C G A G C T A A G C T A G C T G T A C A G T C T G A C | NFkB-p65-Rel(RHD)/ThioMac-LPS-Expression(GSE23622)/Homer | 1e-7 | -1.619e+01 | 0.0000 | 192.0 | 0.97% | 193.5 | 0.65% | motif file (matrix) | svg |
| 143 | C G T A G C T A C G A T C T A G A C G T G T C A C G T A C G T A A G T C C G T A T G C A T A C G | FoxL2(Forkhead)/Ovary-FoxL2-ChIP-Seq(GSE60858)/Homer | 1e-6 | -1.605e+01 | 0.0000 | 1985.0 | 10.05% | 2663.4 | 8.98% | motif file (matrix) | svg |
| 144 | T A C G C T G A C T A G T C G A C G T A A G T C C T G A A T C G G C A T T A G C G A C T A C T G A C G T A G C T A G T C G A C T | GRE(NR),IR3/A549-GR-ChIP-Seq(GSE32465)/Homer | 1e-6 | -1.588e+01 | 0.0000 | 420.0 | 2.13% | 486.7 | 1.64% | motif file (matrix) | svg |
| 145 | C A G T T C A G A G C T G A C T A C G T A G T C G A T C G A C T C T G A A C T G G A T C C G T A C T G A A G T C G T A C | Rfx6(HTH)/Min6b1-Rfx6.HA-ChIP-Seq(GSE62844)/Homer | 1e-6 | -1.583e+01 | 0.0000 | 3438.0 | 17.41% | 4761.6 | 16.05% | motif file (matrix) | svg |
| 146 | C G T A C T G A C T A G C G T A C G T A A G T C C G T A C A G T G C A T G T C A C G A T A C T G A C G T G C A T G A T C | PGR(NR)/EndoStromal-PGR-ChIP-Seq(GSE69539)/Homer | 1e-6 | -1.557e+01 | 0.0000 | 633.0 | 3.21% | 773.1 | 2.61% | motif file (matrix) | svg |
| 147 | T C G A G A C T A G C T T G A C A G C T G T A C T C A G G A T C A T C G T G C A A C T G C T G A | GFX(?)/Promoter/Homer | 1e-6 | -1.509e+01 | 0.0000 | 94.0 | 0.48% | 80.4 | 0.27% | motif file (matrix) | svg |
| 148 | G A C T A G C T G T A C G A C T C T G A A C T G G T C A C T G A A T G C T A C G G A C T A C G T A G T C G A C T C T G A | HRE(HSF)/Striatum-HSF1-ChIP-Seq(GSE38000)/Homer | 1e-6 | -1.463e+01 | 0.0000 | 614.0 | 3.11% | 753.8 | 2.54% | motif file (matrix) | svg |
| 149 | C T G A T C A G A G T C C G T A A T C G A T G C C G A T A C T G A G T C G A C T A T C G A G T C | MyoD(bHLH)/Myotube-MyoD-ChIP-Seq(GSE21614)/Homer | 1e-6 | -1.457e+01 | 0.0000 | 2541.0 | 12.87% | 3479.3 | 11.73% | motif file (matrix) | svg |
| 150 | T C G A A G T C C G T A A T C G A T G C C G A T A C T G A G T C A G C T A C T G | Tcf12(bHLH)/GM12878-Tcf12-ChIP-Seq(GSE32465)/Homer | 1e-6 | -1.426e+01 | 0.0000 | 3030.0 | 15.34% | 4191.2 | 14.13% | motif file (matrix) | svg |
| 151 | T G A C C G A T A C T G A C T G A C T G G A C T A C T G A C G T A C T G A C T G G A T C G A T C | EKLF(Zf)/Erythrocyte-Klf1-ChIP-Seq(GSE20478)/Homer | 1e-5 | -1.375e+01 | 0.0000 | 715.0 | 3.62% | 897.9 | 3.03% | motif file (matrix) | svg |
| 152 | T C A G A T C G G A C T A C T G G A C T C A G T C T A G C G T A G T A C C G T A C T A G A T C G | Tbx20(T-box)/Heart-Tbx20-ChIP-Seq(GSE29636)/Homer | 1e-5 | -1.328e+01 | 0.0000 | 674.0 | 3.41% | 844.2 | 2.85% | motif file (matrix) | svg |
| 153 | G C A T A T C G C A T G G T A C G C T A A G T C T C A G T G A C G T C A T G C A | Arnt:Ahr(bHLH)/MCF7-Arnt-ChIP-Seq(Lo\_et\_al.)/Homer | 1e-5 | -1.316e+01 | 0.0000 | 2036.0 | 10.31% | 2770.3 | 9.34% | motif file (matrix) | svg |
| 154 | C T A G T A C G G A C T T G C A T G C A C G A T T A C G C T G A T C G A C T G A | Hoxa10(Homeobox)/ChickenMSG-Hoxa10.Flag-ChIP-Seq(GSE86088)/Homer | 1e-5 | -1.315e+01 | 0.0000 | 1329.0 | 6.73% | 1761.1 | 5.94% | motif file (matrix) | svg |
| 155 | A G T C C T G A A T C G A G C T A G C T G A C T A G T C G C T A A C G T C G A T G C A T C G A T A T C G C G T A T A G C G C A T A T G C C G T A | bZIP:IRF(bZIP,IRF)/Th17-BatF-ChIP-Seq(GSE39756)/Homer | 1e-5 | -1.306e+01 | 0.0000 | 1298.0 | 6.57% | 1718.9 | 5.79% | motif file (matrix) | svg |
| 156 | A G T C T A G C G A C T A C G T C T A G A C G T A C G T A C G T C T G A A G T C G C T A G A C T C G T A C T A G A C T G | Foxa3(Forkhead)/Liver-Foxa3-ChIP-Seq(GSE77670)/Homer | 1e-5 | -1.280e+01 | 0.0000 | 783.0 | 3.96% | 998.1 | 3.36% | motif file (matrix) | svg |
| 157 | G A C T C T A G C T A G C T A G A C T G T C G A C T G A C T A G C T A G C T A G G T A C G T C A | ZNF467(Zf)/HEK293-ZNF467.GFP-ChIP-Seq(GSE58341)/Homer | 1e-5 | -1.262e+01 | 0.0000 | 3318.0 | 16.80% | 4635.8 | 15.62% | motif file (matrix) | svg |
| 158 | T G C A C G T A G T C A A G C T A G T C G C T A T A G C C G A T C T A G G A T C | Gfi1b(Zf)/HPC7-Gfi1b-ChIP-Seq(GSE22178)/Homer | 1e-5 | -1.232e+01 | 0.0000 | 1743.0 | 8.83% | 2360.8 | 7.96% | motif file (matrix) | svg |
| 159 | C T G A C T A G T G C A C T G A T C G A A G C T C A T G T C G A A G T C G A C T A C G T A G T C G A T C G A T C G A C T | ZNF528(Zf)/HEK293-ZNF528.GFP-ChIP-Seq(GSE58341)/Homer | 1e-5 | -1.214e+01 | 0.0000 | 26.0 | 0.13% | 14.1 | 0.05% | motif file (matrix) | svg |
| 160 | A T G C A G T C G C T A C G A T C G T A G C A T G C T A C G A T C T A G C A T G T G A C G T C A | CArG(MADS)/PUER-Srf-ChIP-Seq(Sullivan\_et\_al.)/Homer | 1e-5 | -1.205e+01 | 0.0000 | 760.0 | 3.85% | 972.3 | 3.28% | motif file (matrix) | svg |
| 161 | T C G A C G T A A G T C A G C T C G T A A G T C T C G A G C T A G A C T C G A T A G T C A G T C A G T C C T G A T C A G T G C A T C G A C A G T A T C G A G T C | GFY-Staf(?,Zf)/Promoter/Homer | 1e-5 | -1.188e+01 | 0.0000 | 284.0 | 1.44% | 326.9 | 1.10% | motif file (matrix) | svg |
| 162 | T C G A T C G A T A C G G A T C G T C A G T A C C G A T A G C T G T C A T G C A | Nkx3.1(Homeobox)/LNCaP-Nkx3.1-ChIP-Seq(GSE28264)/Homer | 1e-5 | -1.169e+01 | 0.0000 | 6747.0 | 34.16% | 9707.7 | 32.72% | motif file (matrix) | svg |
| 163 | A G T C A G T C C G A T A C G T A C G T A C T G A C G T A G C T A G T C A G T C | Sox4(HMG)/proB-Sox4-ChIP-Seq(GSE50066)/Homer | 1e-4 | -1.125e+01 | 0.0000 | 2341.0 | 11.85% | 3236.7 | 10.91% | motif file (matrix) | svg |
| 164 | T A C G T G C A A G T C C G T A A C G T T G A C A C G T A C T G A C T G G C A T | TCF4(bHLH)/SHSY5Y-TCF4-ChIP-Seq(GSE96915)/Homer | 1e-4 | -1.094e+01 | 0.0000 | 4481.0 | 22.69% | 6370.3 | 21.47% | motif file (matrix) | svg |
| 165 | T A C G T C A G T G C A A G C T T G A C A G C T A G T C A C T G G A T C A C T G T C G A A C T G C T G A C T G A A T G C | ZBTB33(Zf)/GM12878-ZBTB33-ChIP-Seq(GSE32465)/Homer | 1e-4 | -1.083e+01 | 0.0001 | 206.0 | 1.04% | 229.5 | 0.77% | motif file (matrix) | svg |
| 166 | A T G C G T A C C G T A A G C T G C A T T A C G A G C T A G C T A G T C A G C T | Sox6(HMG)/Myotubes-Sox6-ChIP-Seq(GSE32627)/Homer | 1e-4 | -1.082e+01 | 0.0001 | 4092.0 | 20.72% | 5800.2 | 19.55% | motif file (matrix) | svg |
| 167 | C T A G C T A G A G T C T C A G A C T G A C G T A C G T C T G A | MYB(HTH)/ERMYB-Myb-ChIPSeq(GSE22095)/Homer | 1e-4 | -1.074e+01 | 0.0001 | 5129.0 | 25.97% | 7330.1 | 24.71% | motif file (matrix) | svg |
| 168 | A T G C T A G C A G C T A G C T T G A C G A C T T C A G T A C G G T C A C T G A A T C G T A G C G A C T C A G T A G T C A G C T T C G A A T C G T G C A T G C A | HRE(HSF)/HepG2-HSF1-ChIP-Seq(GSE31477)/Homer | 1e-4 | -1.043e+01 | 0.0001 | 481.0 | 2.44% | 599.8 | 2.02% | motif file (matrix) | svg |
| 169 | C T A G T C G A T G A C A G T C C G T A A C T G G T A C A C G T A C T G A C T G | BHLHA15(bHLH)/NIH3T3-BHLHB8.HA-ChIP-Seq(GSE119782)/Homer | 1e-4 | -1.041e+01 | 0.0001 | 4128.0 | 20.90% | 5861.8 | 19.76% | motif file (matrix) | svg |
| 170 | A T G C A G T C G A T C C G T A A C G T A C G T A C T G A C G T A G C T G A T C | Sox2(HMG)/mES-Sox2-ChIP-Seq(GSE11431)/Homer | 1e-4 | -1.018e+01 | 0.0001 | 2374.0 | 12.02% | 3300.9 | 11.13% | motif file (matrix) | svg |
| 171 | T C A G T G A C G T A C C G T A A C G T T G A C A C G T T C A G A G C T G A C T | NeuroD1(bHLH)/Islet-NeuroD1-ChIP-Seq(GSE30298)/Homer | 1e-4 | -1.003e+01 | 0.0001 | 2459.0 | 12.45% | 3426.1 | 11.55% | motif file (matrix) | svg |
| 172 | T A G C T C A G C A T G G C A T A G C T C G A T A T G C C G T A C G T A G T C A | CHR(?)/Hela-CellCycle-Expression/Homer | 1e-4 | -9.931e+00 | 0.0001 | 1407.0 | 7.12% | 1908.8 | 6.43% | motif file (matrix) | svg |
| 173 | C A T G T G A C C G T A A G T C T A C G G C A T A C T G G T C A A T G C A G T C | bHLHE41(bHLH)/proB-Bhlhe41-ChIP-Seq(GSE93764)/Homer | 1e-4 | -9.887e+00 | 0.0001 | 3616.0 | 18.31% | 5119.9 | 17.26% | motif file (matrix) | svg |
| 174 | C A G T A C T G T C A G T G C A G C T A A T G C T C G A A T C G G T C A T G C A | ZNF189(Zf)/HEK293-ZNF189.GFP-ChIP-Seq(GSE58341)/Homer | 1e-4 | -9.507e+00 | 0.0002 | 2727.0 | 13.81% | 3825.4 | 12.89% | motif file (matrix) | svg |
| 175 | T C G A G C A T A C G T C T A G G T A C T C G A G C A T T G A C T C G A A C G T | Chop(bZIP)/MEF-Chop-ChIP-Seq(GSE35681)/Homer | 1e-4 | -9.462e+00 | 0.0002 | 428.0 | 2.17% | 533.5 | 1.80% | motif file (matrix) | svg |
| 176 | C T A G C G T A G T C A C G T A A G T C G A T C A G C T C T A G C G T A A C G T G T C A G A T C | Six2(Homeobox)/NephronProgenitor-Six2-ChIP-Seq(GSE39837)/Homer | 1e-4 | -9.301e+00 | 0.0002 | 2549.0 | 12.91% | 3569.9 | 12.03% | motif file (matrix) | svg |
| 177 | T C G A T G A C A G T C C G T A A G T C C T A G A C G T A C T G A C T G A G C T A G T C G C A T | Max(bHLH)/K562-Max-ChIP-Seq(GSE31477)/Homer | 1e-4 | -9.286e+00 | 0.0002 | 2108.0 | 10.67% | 2928.6 | 9.87% | motif file (matrix) | svg |
| 178 | C G T A C T A G T C A G T C A G A G T C A T G C A G T C G C A T A G C T A C G T A T C G C G A T | Sox9(HMG)/Limb-SOX9-ChIP-Seq(GSE73225)/Homer | 1e-3 | -8.947e+00 | 0.0003 | 2411.0 | 12.21% | 3374.2 | 11.37% | motif file (matrix) | svg |
| 179 | C T G A G T A C G A C T A G T C C A G T T G C A C T G A A C G T A G C T G A T C C T A G C G A T A C T G A T G C G A C T C T G A G A T C G A C T A G C T G A T C | Mouse\_Recombination\_Hotspot(Zf)/Testis-DMC1-ChIP-Seq(GSE24438)/Homer | 1e-3 | -8.653e+00 | 0.0004 | 182.0 | 0.92% | 207.8 | 0.70% | motif file (matrix) | svg |
| 180 | A T G C G A T C G A C T A G C T C G A T C G A T G T C A C G A T T C G A A T C G T A G C T A G C | TATA-Box(TBP)/Promoter/Homer | 1e-3 | -8.536e+00 | 0.0005 | 3388.0 | 17.15% | 4811.1 | 16.22% | motif file (matrix) | svg |
| 181 | C G A T C T A G C T G A A T G C C T G A T C G A C G T A C T G A T C G A T A G C A G T C C G T A A C T G T C G A A T G C | Hand2(bHLH)/Mesoderm-Hand2-ChIP-Seq(GSE61475)/Homer | 1e-3 | -8.466e+00 | 0.0005 | 1464.0 | 7.41% | 2009.8 | 6.77% | motif file (matrix) | svg |
| 182 | C T A G A C G T A G T C C G T A A C T G A G T C G C A T A C T G G C A T A G T C G A C T G A T C G C A T A G T C A G C T | ZNF317(Zf)/HEK293-ZNF317.GFP-ChIP-Seq(GSE58341)/Homer | 1e-3 | -8.426e+00 | 0.0005 | 279.0 | 1.41% | 337.6 | 1.14% | motif file (matrix) | svg |
| 183 | G C T A T C G A C G T A C T A G A G C T G T C A G T C A C G T A A G T C C G T A | FOXA1(Forkhead)/LNCAP-FOXA1-ChIP-Seq(GSE27824)/Homer | 1e-3 | -8.410e+00 | 0.0005 | 2939.0 | 14.88% | 4155.5 | 14.01% | motif file (matrix) | svg |
| 184 | C A T G A C G T A G T C G A T C G A T C G A T C G C T A C T A G C T A G C T A G T C A G T C G A | EBF1(EBF)/Near-E2A-ChIP-Seq(GSE21512)/Homer | 1e-3 | -8.384e+00 | 0.0005 | 3296.0 | 16.69% | 4679.8 | 15.77% | motif file (matrix) | svg |
| 185 | T C G A T G A C G T A C C G T A C A G T T G A C A C G T A C T G A G C T A G C T | NeuroG2(bHLH)/Fibroblast-NeuroG2-ChIP-Seq(GSE75910)/Homer | 1e-3 | -8.182e+00 | 0.0007 | 4418.0 | 22.37% | 6336.2 | 21.36% | motif file (matrix) | svg |
| 186 | C G T A C T G A A C T G A G C T A G T C G A T C G A T C G C A T C T G A C T A G C T A G T A C G T C G A T G C A G C A T | EBF2(EBF)/BrownAdipose-EBF2-ChIP-Seq(GSE97114)/Homer | 1e-3 | -8.057e+00 | 0.0007 | 2780.0 | 14.08% | 3929.5 | 13.24% | motif file (matrix) | svg |
| 187 | C T G A T C G A C G T A A T G C C G T A C G T A C G A T C T A G T C A G G A T C | Sox15(HMG)/CPA-Sox15-ChIP-Seq(GSE62909)/Homer | 1e-3 | -7.775e+00 | 0.0010 | 2738.0 | 13.86% | 3873.7 | 13.06% | motif file (matrix) | svg |
| 188 | C T G A C T A G A T C G G C A T A C T G G T A C A T G C C G T A A C T G G C T A A G T C C G T A | Tbox:Smad(T-box,MAD)/ESCd5-Smad2\_3-ChIP-Seq(GSE29422)/Homer | 1e-3 | -7.774e+00 | 0.0010 | 570.0 | 2.89% | 743.9 | 2.51% | motif file (matrix) | svg |
| 189 | C G A T C T A G A C G T A C G T A C G T C G T A A G C T C G A T A G C T C G T A C T A G T A G C | FoxD3(forkhead)/ZebrafishEmbryo-Foxd3.biotin-ChIP-seq(GSE106676)/Homer | 1e-3 | -7.768e+00 | 0.0010 | 2309.0 | 11.69% | 3246.4 | 10.94% | motif file (matrix) | svg |
| 190 | A C G T T C G A T C G A A G T C G T C A T A C G A T G C A C G T A C T G A G C T | Myf5(bHLH)/GM-Myf5-ChIP-Seq(GSE24852)/Homer | 1e-3 | -7.756e+00 | 0.0010 | 2136.0 | 10.82% | 2994.8 | 10.09% | motif file (matrix) | svg |
| 191 | C T A G T A C G G A T C G T C A T G C A A C G T T G C A G C T A T C G A T G C A | Hoxa9(Homeobox)/ChickenMSG-Hoxa9.Flag-ChIP-Seq(GSE86088)/Homer | 1e-3 | -7.720e+00 | 0.0010 | 6373.0 | 32.27% | 9247.7 | 31.17% | motif file (matrix) | svg |
| 192 | T A C G A T G C G C T A A C T G C G T A A C G T C G T A C T G A T A C G T C G A | Gata4(Zf)/Heart-Gata4-ChIP-Seq(GSE35151)/Homer | 1e-3 | -7.717e+00 | 0.0010 | 2283.0 | 11.56% | 3209.2 | 10.82% | motif file (matrix) | svg |
| 193 | T G A C T C G A C T G A C T G A A T G C G A T C C T A G T A C G G A C T G A C T G A T C T C G A C T G A C T G A A T G C G A T C C T A G A T C G G A C T G A C T | Tcfcp2l1(CP2)/mES-Tcfcp2l1-ChIP-Seq(GSE11431)/Homer | 1e-3 | -7.593e+00 | 0.0011 | 531.0 | 2.69% | 690.3 | 2.33% | motif file (matrix) | svg |
| 194 | C T A G C A G T T G A C C G T A G A T C T C A G G A C T C A T G | BMAL1(bHLH)/Liver-Bmal1-ChIP-Seq(GSE39860)/Homer | 1e-3 | -7.527e+00 | 0.0012 | 5058.0 | 25.61% | 7299.1 | 24.60% | motif file (matrix) | svg |
| 195 | T G A C G C T A T G A C C G T A T C A G G A T C C G T A C A T G C A T G C T A G C T A G C T A G | Unknown-ESC-element(?)/mES-Nanog-ChIP-Seq(GSE11724)/Homer | 1e-3 | -7.492e+00 | 0.0013 | 1880.0 | 9.52% | 2626.3 | 8.85% | motif file (matrix) | svg |
| 196 | A C G T C T A G C G T A A G T C G T A C A C G T A C G T A C G T G T C A G T A C T G A C G A C T | Nur77(NR)/K562-NR4A1-ChIP-Seq(GSE31363)/Homer | 1e-3 | -7.481e+00 | 0.0013 | 414.0 | 2.10% | 528.6 | 1.78% | motif file (matrix) | svg |
| 197 | T C A G G C T A T C A G C A G T T G A C G T C A A G T C A T C G T G C A G T A C C A G T G A T C | Npas4(bHLH)/Neuron-Npas4-ChIP-Seq(GSE127793)/Homer | 1e-3 | -7.450e+00 | 0.0013 | 2305.0 | 11.67% | 3246.7 | 10.94% | motif file (matrix) | svg |
| 198 | C T A G A G C T G A C T C A T G A G T C A G T C G T C A C A G T C T A G T C A G G T A C C T G A T C G A G A T C T G A C | Rfx2(HTH)/LoVo-RFX2-ChIP-Seq(GSE49402)/Homer | 1e-3 | -7.259e+00 | 0.0016 | 328.0 | 1.66% | 412.0 | 1.39% | motif file (matrix) | svg |
| 199 | G C T A T C G A C G T A C T A G A G C T G T C A G T C A C G T A A G T C C G T A | FOXA1(Forkhead)/MCF7-FOXA1-ChIP-Seq(GSE26831)/Homer | 1e-3 | -7.141e+00 | 0.0018 | 2379.0 | 12.05% | 3360.3 | 11.33% | motif file (matrix) | svg |
| 200 | T G A C C T G A C T A G T C G A C T G A A T G C C G T A A C T G G C A T G T A C G C A T A T C G G C A T A G C T G A T C | PR(NR)/T47D-PR-ChIP-Seq(GSE31130)/Homer | 1e-3 | -7.052e+00 | 0.0019 | 6525.0 | 33.04% | 9492.9 | 32.00% | motif file (matrix) | svg |
| 201 | C A T G A C T G C T A G T C G A T C G A T C G A T C G A T C A G T C A G T C A G T G A C T G A C C G T A A C T G T G C A C G A T A C T G | RBPJ:Ebox(?,bHLH)/Panc1-Rbpj1-ChIP-Seq(GSE47459)/Homer | 1e-2 | -6.676e+00 | 0.0028 | 1000.0 | 5.06% | 1366.6 | 4.61% | motif file (matrix) | svg |
| 202 | T A G C G C T A A C T G C G T A A C G T C G T A C G T A T A C G T C A G T C G A | Gata1(Zf)/K562-GATA1-ChIP-Seq(GSE18829)/Homer | 1e-2 | -6.658e+00 | 0.0028 | 1346.0 | 6.82% | 1865.8 | 6.29% | motif file (matrix) | svg |
| 203 | C T G A T G A C T G A C C G T A A C G T T G A C A G C T C T A G A C G T G A C T | Olig2(bHLH)/Neuron-Olig2-ChIP-Seq(GSE30882)/Homer | 1e-2 | -6.631e+00 | 0.0029 | 5436.0 | 27.52% | 7884.2 | 26.57% | motif file (matrix) | svg |
| 204 | T C G A G C A T A T G C C T G A A T G C T A G C A G T C G T A C T C G A A G C T | Srebp1a(bHLH)/HepG2-Srebp1a-ChIP-Seq(GSE31477)/Homer | 1e-2 | -6.451e+00 | 0.0034 | 520.0 | 2.63% | 685.6 | 2.31% | motif file (matrix) | svg |
| 205 | A G C T A G T C A T G C A G C T A C G T C G T A A C G T A G T C C G A T A T G C | Gata2(Zf)/K562-GATA2-ChIP-Seq(GSE18829)/Homer | 1e-2 | -5.991e+00 | 0.0054 | 1504.0 | 7.62% | 2105.3 | 7.10% | motif file (matrix) | svg |
| 206 | A G T C A T C G C T A G A G C T G A C T C T A G A G T C A G T C G C T A C A G T T C A G T C A G G A T C C T G A T C G A G A T C | RFX(HTH)/K562-RFX3-ChIP-Seq(SRA012198)/Homer | 1e-2 | -5.977e+00 | 0.0054 | 301.0 | 1.52% | 383.6 | 1.29% | motif file (matrix) | svg |
| 207 | C T G A C T A G T C G A C G T A A T G C C G T A A T C G C G A T T A G C G C A T A T C G G C A T A G C T G A T C G A C T A G C T | ARE(NR)/LNCAP-AR-ChIP-Seq(GSE27824)/Homer | 1e-2 | -5.948e+00 | 0.0055 | 824.0 | 4.17% | 1123.4 | 3.79% | motif file (matrix) | svg |
| 208 | C T G A C A T G A C T G A C G T A T G C C G T A C A T G T A C G A T G C G C T A T A C G C T G A C T A G A C T G A C G T A T G C C G T A T A G C | RAR:RXR(NR),DR5/ES-RAR-ChIP-Seq(GSE56893)/Homer | 1e-2 | -5.920e+00 | 0.0057 | 83.0 | 0.42% | 90.1 | 0.30% | motif file (matrix) | svg |
| 209 | A C G T C T G A G A T C A T C G G A C T T C A G G T A C T A G C | HIF-1a(bHLH)/MCF7-HIF1a-ChIP-Seq(GSE28352)/Homer | 1e-2 | -5.906e+00 | 0.0057 | 716.0 | 3.63% | 969.4 | 3.27% | motif file (matrix) | svg |
| 210 | G T A C G A T C C A G T A G T C A G T C A G T C T G C A G A T C C T G A A T G C G T C A A C G T | WT1(Zf)/Kidney-WT1-ChIP-Seq(GSE90016)/Homer | 1e-2 | -5.634e+00 | 0.0075 | 2276.0 | 11.52% | 3240.6 | 10.92% | motif file (matrix) | svg |
| 211 | G C T A C G A T C A T G A T G C A G T C A G T C G A C T T A C G T C G A C T A G A C T G T A G C | AP-2alpha(AP2)/Hela-AP2alpha-ChIP-Seq(GSE31477)/Homer | 1e-2 | -5.531e+00 | 0.0083 | 2716.0 | 13.75% | 3889.8 | 13.11% | motif file (matrix) | svg |
| 212 | T A G C G C A T A G T C G A T C A T G C G A C T C T A G A C T G A C T G C T G A A C T G C T A G A G T C T G A C C G A T | GLIS3(Zf)/Thyroid-Glis3.GFP-ChIP-Seq(GSE103297)/Homer | 1e-2 | -5.496e+00 | 0.0085 | 4801.0 | 24.31% | 6974.8 | 23.51% | motif file (matrix) | svg |
| 213 | T C G A T C G A A G T C C G T A C T A G T A G C A C G T A C T G | MyoG(bHLH)/C2C12-MyoG-ChIP-Seq(GSE36024)/Homer | 1e-2 | -5.431e+00 | 0.0090 | 3263.0 | 16.52% | 4698.3 | 15.84% | motif file (matrix) | svg |
| 214 | A G C T A T G C G A C T G C A T C G T A A G C T G T A C C G A T A T C G A G T C | Gata6(Zf)/HUG1N-GATA6-ChIP-Seq(GSE51936)/Homer | 1e-2 | -5.396e+00 | 0.0093 | 2027.0 | 10.26% | 2880.4 | 9.71% | motif file (matrix) | svg |
| 215 | G A C T C T A G A T G C A G T C G T C A T A C G A T G C A T C G | HIC1(Zf)/Treg-ZBTB29-ChIP-Seq(GSE99889)/Homer | 1e-2 | -5.379e+00 | 0.0094 | 6407.0 | 32.44% | 9368.5 | 31.58% | motif file (matrix) | svg |
| 216 | T A G C G T A C A G T C G T A C C G A T A G T C A G T C A G T C A G T C A G T C C G T A G A T C | Zfp281(Zf)/ES-Zfp281-ChIP-Seq(GSE81042)/Homer | 1e-2 | -5.366e+00 | 0.0095 | 1064.0 | 5.39% | 1477.8 | 4.98% | motif file (matrix) | svg |
| 217 | T A C G C T G A T C G A C G A T C T A G C T A G T C G A C T G A T C G A T C G A C G T A T C G A G C A T C A T G C G T A T A C G G C A T T G A C C G T A A G C T | NFAT:AP1(RHD,bZIP)/Jurkat-NFATC1-ChIP-Seq(Jolma\_et\_al.)/Homer | 1e-2 | -5.348e+00 | 0.0096 | 411.0 | 2.08% | 542.3 | 1.83% | motif file (matrix) | svg |
| 218 | C T A G T A C G G A T C G T C A T C G A A C G T T C A G C G T A C G T A C G T A | Hoxd10(Homeobox)/ChickenMSG-Hoxd10.Flag-ChIP-Seq(GSE86088)/Homer | 1e-2 | -5.222e+00 | 0.0109 | 2643.0 | 13.38% | 3789.6 | 12.77% | motif file (matrix) | svg |
| 219 | T C A G C T A G A T G C G A C T T G C A C G T A A G C T A C G T C T G A T A C G | En1(Homeobox)/SUM149-EN1-ChIP-Seq(GSE120957)/Homer | 1e-2 | -5.119e+00 | 0.0120 | 4083.0 | 20.67% | 5920.3 | 19.95% | motif file (matrix) | svg |
| 220 | A T G C G A T C C G T A A G C T C A G T A T C G G C A T A G C T G A C T A C T G | Sox17(HMG)/Endoderm-Sox17-ChIP-Seq(GSE61475)/Homer | 1e-2 | -5.066e+00 | 0.0126 | 1758.0 | 8.90% | 2493.5 | 8.40% | motif file (matrix) | svg |
| 221 | T C A G T A C G G A T C G T A C T C G A C G A T C T G A C G T A G C T A C G T A | Hoxd11(Homeobox)/ChickenMSG-Hoxd11.Flag-ChIP-Seq(GSE86088)/Homer | 1e-2 | -4.767e+00 | 0.0169 | 5553.0 | 28.12% | 8116.2 | 27.36% | motif file (matrix) | svg |
| 222 | C T G A T C A G G T A C T G C A A G T C C G T A A G T C A C T G A C G T A C T G | MNT(bHLH)/HepG2-MNT-ChIP-Seq(Encode)/Homer | 1e-2 | -4.759e+00 | 0.0170 | 2978.0 | 15.08% | 4295.3 | 14.48% | motif file (matrix) | svg |
| 223 | C A G T G A C T A G T C G C T A C T G A G T C A C G T A A G C T G T C A C T G A C T G A C T G A A C T G A G C T G A T C | MYNN(Zf)/HEK293-MYNN.eGFP-ChIP-Seq(Encode)/Homer | 1e-2 | -4.727e+00 | 0.0175 | 557.0 | 2.82% | 756.7 | 2.55% | motif file (matrix) | svg |
| 224 | A C T G A C G T C A T G A T C G A T C G T G A C A C T G A T C G A T C G T G C A C T G A C G T A | E2F3(E2F)/MEF-E2F3-ChIP-Seq(GSE71376)/Homer | 1e-2 | -4.701e+00 | 0.0178 | 2345.0 | 11.87% | 3363.5 | 11.34% | motif file (matrix) | svg |
| 225 | C T G A A C T G A C T G A G T C A G T C A G C T C T A G T A C G | ZFX(Zf)/mES-Zfx-ChIP-Seq(GSE11431)/Homer | 1e-2 | -4.628e+00 | 0.0191 | 4114.0 | 20.83% | 5981.0 | 20.16% | motif file (matrix) | svg |
| 226 | T C A G C A T G C A T G A C T G A C T G A G C T A C T G A C G T A C T G C A G T A T G C A G T C | KLF10(Zf)/HEK293-KLF10.GFP-ChIP-Seq(GSE58341)/Homer | 1e-2 | -4.622e+00 | 0.0191 | 2474.0 | 12.53% | 3555.9 | 11.99% | motif file (matrix) | svg |
